# Robotic surgery in Asia

**DOI:** 10.1016/j.amsu.2021.102890

**Published:** 2021-09-30

**Authors:** Safinaz Khan, A.H.M. Ataullah, Robert Ahmed Khan, Mohammed Maan Al-Salihi, Sabrina Rahman, Md Moshiur Rahman

**Affiliations:** a Department of Biochemistry and Molecular Biology, Sir Salimullah Medical College, Dhaka, Bangladesh; b Medical Officer, Sher-E-Bangla Medical College Hospital, Barishal, Bangladesh; c Medical Officer, Neurosurgery Department, Bangabandhu Sheikh Mujib Medical University, Dhaka, Bangladesh; d Medical Doctor, College of Medicine, University of Baghdad, Baghdad, Iraq; e Department of Public Health, Independent University-Bangladesh, Dhaka, Bangladesh; f Neurosurgery Department, Holy Family Red Crescent Medical College, Dhaka, Bangladesh

## Abstract

The percentage of robot-assisted radical cystectomy (RARC) is comparatively low in Asia compared to other continents [11]. Few studies showed, RARC with intracorporeal urinary diversion causes less bleeding and hospital stay compared with extracorporeal urinary diversion.

Robotic surgery has largely replaced traditional laparoscopic surgery. These new emerging techniques have been highly practicing in China, Korea, and Japan among other Asian countries, are being incorporated in more complex surgeries [1]. These techniques can be applied for cancer treatment as well as infectious disease also. Robots can perform surgeries from head to anywhere in the human body, but robotic surgery of the gastrointestinal tract is playing a role model for improvement in treatment and opening a new gate for research scope. Robotic surgery using da Vinci Surgical System-a robot-assisted minimal invasive surgery, which has high-resolution 3-dimensional images and seven degrees of robotic arms, approved by the Food and Drug Administration (FDA) in 2000 [2].

Based on a meta-analysis, during colorectal surgery, robotic-assisted colorectal surgery (RACS) showed a lowered level of estimated blood loss (EBL), early postoperative morbidity, and length of hospital stay (LHS) than laparoscopic-assisted colorectal surgery (LACS). The rate of complication and resection accuracy according to oncology were assessed, showing almost similar results. Besides treating regular cases, a rare vascular disease, Diffuse cavernous haemangioma of the rectum (DCHR) is seen to be treated successfully by robot-assisted resection [3].

Since gastrectomy is the cornerstone of gastric cancer treatment, patient compliance is a major concern. Laparoscopic distal gastrectomy (LDG) is a gold standard treatment for patients with advanced gastric cancer (stage 1–3). Even though operation time is longer in robot-assisted distal gastrectomy (RADG), comparing with 3D-laparoscopic assisted 2D radical distal gastrectomy (3D-LADG), the earlier group is more convenient with patients in case of cost and hospital stay, but overall complications and efficacies are similar in both groups [4]. A new milestone for gastric carcinoma is intraoperative imaging during robotic surgery. Robotic gastrectomy has overcome some drawbacks also related to laparoscopic gastrectomy, for example; limited movements around the peripancreatic region, hand trembling. In the case of gall bladder cancer, robot extended cholecystectomy (REC) is favorable because of retrieval of some lymph nodes associated with early recovery [5]. Robotic pancreatic enucleation has been proven an efficient procedure alternative to open pancreatic enucleation used for benign or borderline pancreatic cancer. Similarly, Robotic distal pancreatectomy (RDP) is also regarded as a viable alternative to laparoscopic distal pancreatectomy (LDP) with better spleen and splenic vessel preservation (SVP) in medium tumors [6,7].

In pediatrics, da Vinci surgical system consists of Roux-en-Y limb formation, showed feasibility in the case of choledochal cyst excision and hepaticojejunostomy. Intraoperative and postoperative complications were found less in robot-assisted surgery than laparoscopic surgery [8]. Achalasia, comparatively rare in children can be cured with no post-operative complication by robot-assisted Heller's myotomy via da Vinci surgical system [9].

Robot-assisted surgical atrial fibrillation ablation study on a small number of patients showed high survival with low mortality rate, where no patients required permanent pacemaker implantation [10].

Radio-chemotherapy is the key treatment for early oropharyngeal cancer but in advanced cases, minimally invasive procedure, transoral robotic surgery (TORS) is the treatment choice, which has been proven with better oncological outcome in oropharyngeal cancers [12,13]. In HPV negative supraglottic carcinoma, TORS is recommended as this minimally invasive procedure can avoid complications resulting from radiotherapy with less morbidity and early recovery [14].

Transoral robotic thyroidectomy (TORT)-a scar-free surgery is widely popular and most cases are reported in Asia. TORT is applicable in any thyroid disorders like a benign nodule, papillary, and follicular carcinoma irrespective of size or lobectomy. This procedure reported no temporary and permanent vocal cord palsy or recurrence with no mortality [15].

In most studies, the main obstacles of robotic surgery are the high expenses and time consumption. Patient benefits are also unrecognized. According to some studies, there are some limitations due to lack of precision, such as vessel ligation, inspecting body cavity, and longer learning curve [16]. Robotic surgery needs to perform cautiously - so many instruments are intercalated and operators must be skillful to provide the best outcome.

This modern approach has encountered the biggest challenge-incorporating humans and instruments simultaneously. Furthermore, operating and maintenance of this high-end machinery system are one of the disputes. Cost is regarded as a hurdle but we can anticipate this is a matter of time to reduce the pricing when robotic surgery will be more popular, full-blown, and widely available.

Despite mentioned drawbacks, there are benefits also. Robotic surgery takes the upper hand to have the 3D visualization with 7° wrist-like motion, flexibility, no exhaustion, tremor refined, motion scaling, and avoidance of fulcrum effect, associated with laparoscopic surgery [17]. Robotic procedure accompanied by new methods-fluorescence in situ, virtual reality software, picture in picture technology, and EndoWrist manipulation [18].

Robotic surgery has already taken over some surgical fields and setting new goals every day. By this procedure, a surgeon can operate remotely and this process aid to reduce human workload. Scientists are working dedicatedly to improve this promising field. This emerging technology can move into a whole new era with advanced technology and further exploration in surgical sectors.

## Ethical approval

It is not necessary.

## Sources of funding

None.

## Author contribution

All authors equally contributed to the analysis and writing of the manuscript.

## Research registration Unique Identifying number (UIN)

1 Name of the registry: Not applicable.
2 Unique Identifying number or registration ID: Not applicable.
3 Hyperlink to your specific registration (must be publicly accessible and will be checked): Not applicable.

## Guarantor

Md Moshiur Rahman, Assistant Professor, Neurosurgery Department, Holy Family Red Crescent Medical College, Dhaka, Bangladesh. Email: dr.tutul@yahoo.com.

## Declaration of competing interest

None.
